# A highly sensitive uric acid electrochemical biosensor based on a nano-cube cuprous oxide/ferrocene/uricase modified glassy carbon electrode

**DOI:** 10.1038/s41598-020-67394-8

**Published:** 2020-06-30

**Authors:** Qinghua Yan, Na Zhi, Li Yang, Guangri Xu, Qigao Feng, Qiqing Zhang, Shujuan Sun

**Affiliations:** 1 The Key Laboratory of Biomedical Material, School of Life Science and Technology, Xinxiang Medical University, Xinxiang, 453003 China; 2 School of Chemistry and Chemical Engineering, Henan Institute of Science and Technology, Xinxiang, 453003 China; 3 Institute of Biomedical Engineering, Chinese Academy of Medical Sciences and Peking Union Medical College, Tianjin, 300192 China; 4 The Hospital of Eighty-Third Group Army, Xinxiang, 453000 China

**Keywords:** Analytical biochemistry, Nanobiotechnology, Sensors and probes

## Abstract

A uric acid (UA) electrochemical biosensor was constructed using ferrocene (Fc) decorated cuprous oxide (Cu_2_O) enhanced electro-active characteristics and covalently immobilized with uricase (UOx) on glassy carbon electrode (GCE). The electrochemical characteristics of the fabricated electrode was analysed by cyclic voltammetry, electrochemical impedance spectroscopy and differential pulse voltammetry (DPV). DPV studies revealed rapid response of fabricated electrode UOx/Fc/Cu_2_O/GCE towards UA in a wide concentration range of 0.1–1,000 μM with a sensitivity of 1.900 μA mM^−1^ cm^−2^ and very low detection limit of 0.0596 μM. A very low magnitude Michaelis–Menten constant (Km) value was evaluated as 34.7351 μM which indicated the chemical attraction of the enzyme towards the UA was much higher. The developed biosensor was successfully applied to detect UA in human urine samples. Moreover, reproducibility and stability studies demonstrated the fabricated UOx/Fc/Cu_2_O/GCE biosensor had high reproducibility with a RSD of 2.8% and good reusability with a RSD of 3.2%. Specificity studies results showed the fabricated biosensor had strong anti-interference ability. The improved sensor performance was attributed to the synergistic electronic properties of Cu_2_O and Fc that provided enhances delectrocatalytic activity and electron transfer. The present biosensor can be extended for use in clinical settings.

## Introduction

In the current worldwide field of disease diagnosis and treatment, there is an urgent requirement to develop a device that can recognize, detect and quantify metabolites in important biochemical processes of human body for rapid, simple and accurate diagnosis of various diseases. Uric acid, as an antioxidant, exists in urine or serum excreted by the kidney and is the end-product of purine nucleotide metabolism and its derivatives and reacts to many biological changes in the human body.^1,2^ The increased concentration in body fluids from its normal range^3^ is associated with several medical conditions such as gout,^4^ hyperuricemia, Lesch–Nyhansyndrome,^5^ kidney disease and cardiovascular disease.^1^ Therefore, it becomes imperative to monitor UA levels regularly. The commonly used methods to detect UA in clinic include high performance liquid chromatography (HPLC),^6^ spectrophotometry^7^ chemiluminescence analysis, enzyme electrode analysis, uric acid biosensor,^8,9^ and so on. Among them, uric acid biosensor has been paid more and more attention because of its advantages of simple manufacture, low cost, high sensitivity and good selectivity.^10–12^

UOx (urate oxidase) catalyzes the oxidation reaction of UA to produce allantoin during the degradation of purine. Due to the selectivity and high specificity of UOx toward UA, UOx has been proved to be very effective for biosensor fabrication.^13^ The first approach used for transducing biochemical reaction to electrical signal for redox enzyme-based amperometric biosensors is detection of redox active co-substrates or co-products. The second approach is an electron-transfer mediators are used to exchange electrons between the active center of the enzyme and the electrode.^14,15^ However, the low enzyme activity, sluggish electrode kinetics and high over potential resulting in reduced charge transfer and increased interference from other electroactive substances coexisting with the sample and at the same time suffer from leaching of soluble mediators from the electrode surface still present some of the major drawbacks of devices.^16^ Based on the above two approaches. The third biosensing systems that the electrons involved in the redox process are shuttled directly between the redox centre of the enzyme and the electrode surface to generate the response are very promising due to high stability and mediator independent operation and interference free detection of analytes. However, the main obstacle for the development of biosensors is the unreachable redox centers in many redox enzyme proteins, which hinder the electronic flow between the enzyme and the electrodes. The addition of highly conductive nanomaterials to establish bioelectrode is becoming a selective tool for direct electron transfer biosensing systems.

Nanomaterials have large surface-to-volume ratio and good electron transfer ability.^17^ The electrocatalytic performance of nanomaterials depends not only on the size of materials and the composition of elements, but also on the morphology of materials.^18^ As a new type of P-type oxide semiconductor material, Cu_2_O nanocrystal possesses active electron–hole system, which exhibits good electrocatalytic activity.^19^ Khan et al. fabricated a glucose biosensor using shuriken-like Cu_2_O nanoparticles (Cu_2_O NPs) and discovered that Cu_2_O showed good electrocatalytic effect.^20^

Ferrocene (Fc) has stable chemical properties and low toxicity, and has excellent redox reversibility, which can react with the active center of biological enzymes and rapidly improve the density of response current, so Fc and its derivatives are widely used as electronic mediators.^21^ Conjugation of Fc with biomolecules such as DNA, amino acids and peptides can provide novel systems promoting electron transfer.^22^ However, Fc does not adsorb well on the electrode surface, especially its cation (Fc^+^) is easy to lose in electrolyte, so the stable existence of Fc on the electrode is particularly important. Nafion is a perfluorosulfonic acid polymer solution that contains sulfonic acid group, which can generate electrostatic adsorption to the cationic form of Fc, so that Fc can be stable in the form of cation on the electrode.^23^ The electron transfer rate can be greatly improved by adding Fc to modified electrode as an electronic medium. A Nafion supported Fc-modified electrode was successfully used by Chen et al. in sensor development.^24^

In the present study, we reported a novel enzymatic electrochemical biosensor for sensitive and selective detection of UA using UOx with enhanced biocatalytic activity on the electrode surface. The UOx with enhanced activity was immobilized on a Naf platform in a Cu_2_O nanomaterial and electronic mediator Fc on the electrode surface. The electrochemical characteristics of the fabricated electrode was analysed by cyclic voltammetry (CV) and electrochemical impedancespectroscopy (EIS). The response behaviours of the modified electrode with various UA concentrations were investigated by differential pulsevoltammetry (DPV). The analytical performance of the modified electrode toward UA detection was also demonstrated using the modified electrode.

## Experimental section

### Chemicals and reagents

Uric acid, uricase, ferrocene, Nafion 117 (Naf; 5 wt.%), potassium hexacyanoferrate (III) (K_3_Fe(CN)_6_) and potassium hexacyanoferrate(II) trihydrate (K_4_Fe(CN)_6_·3H_2_O), were purchased from Sigma Aldrich. BSA, ascorbic acid (AA), glucose (Glu), urea, and cysteine (Cys) were supplied by Sangon Biotech Co.,Ltd. (Shanghai, China).Copper (II) chloride (CuCl_2_) was purchased from Aladdin Biochemical Technology Co., Ltd (Shanghai, China). A 100 mM phosphate buffer saline (PBS) solution was prepared from NaH_2_PO_4_, Na_2_HPO_4_ and NaCl came from Merck. All the chemicals were of analytical grade and purchased from Sigma Aldrich. All the reagent solutions and buffer solutions were prepared with Milli-Q water (18.2 MΩ). Healthy urine samples for analysis were collected from the Third Affiliated Hospital of Xinxiang Medical University. All the methods are carried out in relevant guidelines and regulations. The study protocol is approved by the Ethics Committee of Xinxiang Medical University and the Henan Ministry of Health and Medical Services. All the participants submitted the written informed consent for the study.

### Synthesis and characterization of Cu_2_O NPs

Cu_2_O NPs were synthesized as per the previous method^19^ with few modifications. Add 1.0 mL 0.1 M CuCl_2_ solution and 1.0 mL 0.5 M NaOH solution to 50 mL ultra-pure water. The mixture solution was continuously stirred until the blue Cu(OH)_2_ precipitation appeared. After the mixture solution being stirred constantly for 5 min, 1.0 mL 0.1 M solution of ascorbic acid was added into the mixture. The color of precipitation changed from blue to yellow and finally to brick red. After the reaction being completed for 30 min, The precipitates were centrifuged at 10,000×*g* for 5 min, washed with ultra-pure water for 3 times, and then vacuum dried at 60 °C for 5 h. The products dried (Cu_2_O NPs) were kept in absolute ethyl ethanol for subsequent experiments.

The structural analysis of synthesized Cu_2_O NPs was carried out using X-Ray diffractometer (XRD; BRUKER.axs, Germany) and Transmission Electron Microscopy (TEM; HT7700, Hitachi High-Technologies, Japan). The electronic states of the samples surface were analyzed by the X-ray photoelectron spectroscopy (XPS) at VG ESCALAB MK II instrument with an Mg Ka (1,253.6 eV) achromatic.

### Preparation and characterization of differently modified electrodes

The bare glassy carbon electrode was polished with Al_2_O_3_ powder (0.05 μm), and then ultrasonic washed with 1:1 nitric acid solution, ethanol 70% (v/v), distilled water and Milli-Q water successively. The glassy carbon electrode was scanned and activated to stability by cyclic voltammetry in 0.5 M sulfuric acid, and the activated glassy carbon electrode was naturally dried at room temperature for later use.

**Figure 1:**
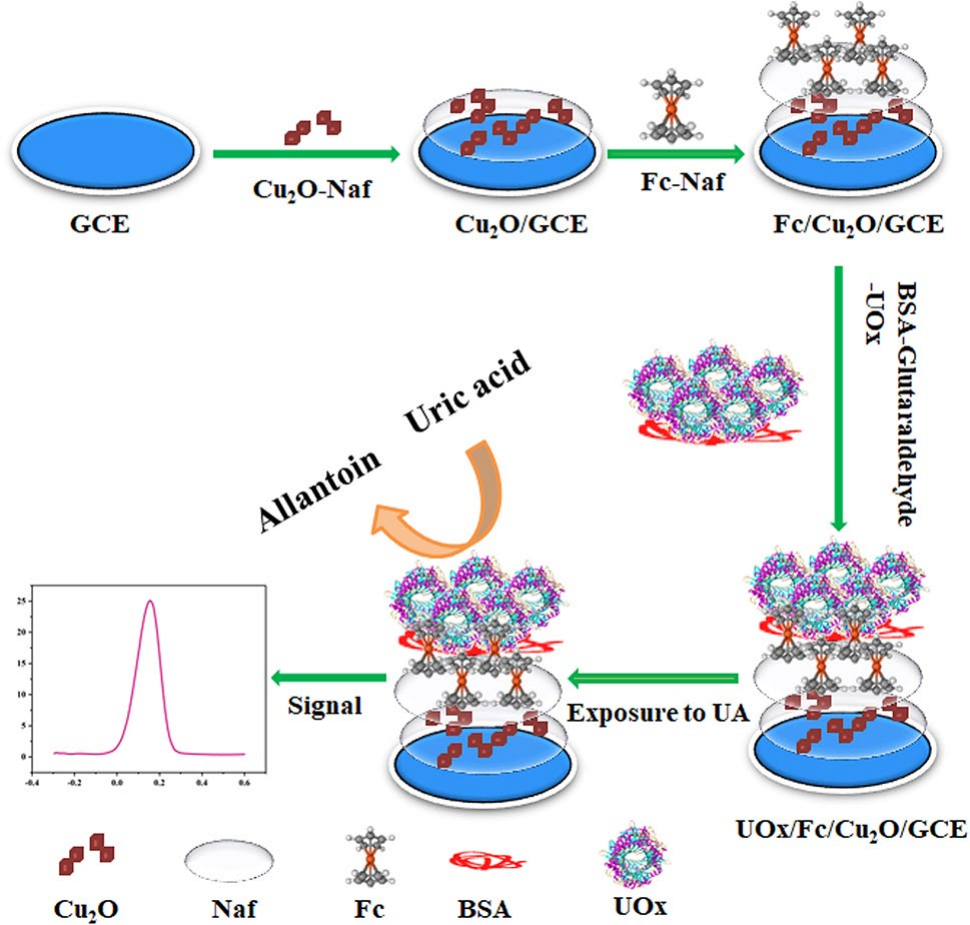
The fabrication process of biosensor and UA detection.

5 µL of the Cu_2_O-Naf mixture (200 µL 8 mg mL^−1^ Cu_2_O dispersed in 200 µL 5% Naf solution) was coated on the GCE surface and allowed to dry under clean air at room temperature. Then GCE was dropped with 6 µL 10 mg mL^−1^ Fc-Naf mixture (1 mg Fc was dissolved in 50 µL ethanol and 50 µL 5% Naf solution) and dried at room temperature. Finally 3 µL mixture solution containing UOx (10 mg L^−1^), BSA (8.0 mg L^−1^), and glutaraldehyde (1.25%) was immobilized on the modified GCE surface. The modified GCE was rinsed with PBS to remove excess reactant after being dried for 12 h at 4 °C, and stored in PBS at 4 °C for use. The schematic for the fabrication of electrode was shown in Fig. 1.

The characterization of differently modified electrodes (i.e. Cu_2_O/GCE, Fc/Cu_2_O/GCE, UOx/Fc/Cu_2_O/GCE) were determined by FEI scanning electron microscope (SEM) (model Quanta-200, Netherlands) with Oxford energy dispersive X-ray spectrometry (EDS) (model INCA 250 x, England).

### Electrochemical measurements of the modified electrode

CV, EIS and DPV techniques were using to investigate the electrochemical characterization and analytical performance of the modified electrode.

## Results and discussion

### Characterization of Cu_2_O NPs

**Figure 2:**
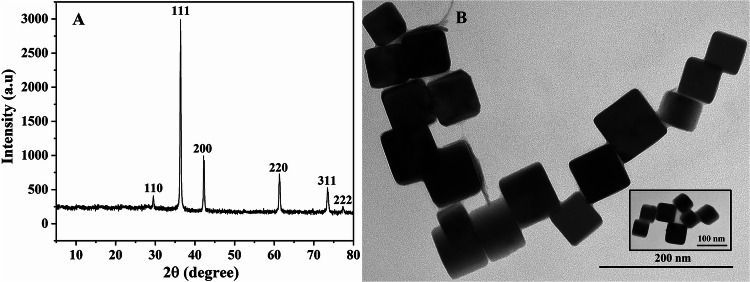
(**A**) Powder x-ray diffraction patterns of Cu_2_O NPs. (**B**) TEM micrographs of Cu_2_O NPs.

The structure and morphology of the synthesized Cu_2_O NPs were detected by different characterization tools (Fig. 2). XRD spectra of Cu_2_O NPs showed the diffraction peaks appeared at a 2θ value of 29.48°, 36.38°, 42.20°, 61.32°, 75.26° and 76.30° corresponds to the (110), (111), (200), (220), (311) and (222) characteristic planes, and confirmed that all the diffraction peaks in the XRD spectrum correspond to the standard pattern of cuprous oxide cubic crystal system (PDF#05-0667, cell parameter a = 0.4269 nm) (Fig. S1), and had high diffraction intensity and sharp peak shape, indicating that the products had good crystallinity^25,26^. It can also be seen from the XRD spectra that the synthesized nanoparticles had no impurity peak, indicating that the product had a single crystal shape and high crystal purity (Fig. 2A). TEM images revealed the prepared Cu_2_O was cubic, with good dispersibility, uniform size and formation of highly crystalline Cu_2_O NPs with the size of about 60 nm (Fig. 2B). XPS was further used to analyze the surface elemental composition and chemical environment. The full XPS spectrum of Cu_2_O showed that the sample contain Cu and O elements. In the high resolution XPS spectrum of Cu_2_O, there were two characteristic peaks at 951.9 eV and 932.1 eV can be referred to Cu^+^ (Fig. S2A).^27,28^ There were also two characteristic peaks at 530.1 eV and 533.2 eV respectively, which belonged to Cu–O bond and H_2_O adsorbed on the surface (Fig. S2B),^29,30^ indicating that the freshly prepared Cu_2_O was relatively stable.

### Characterization of differently modified electrodes

**Figure 3:**
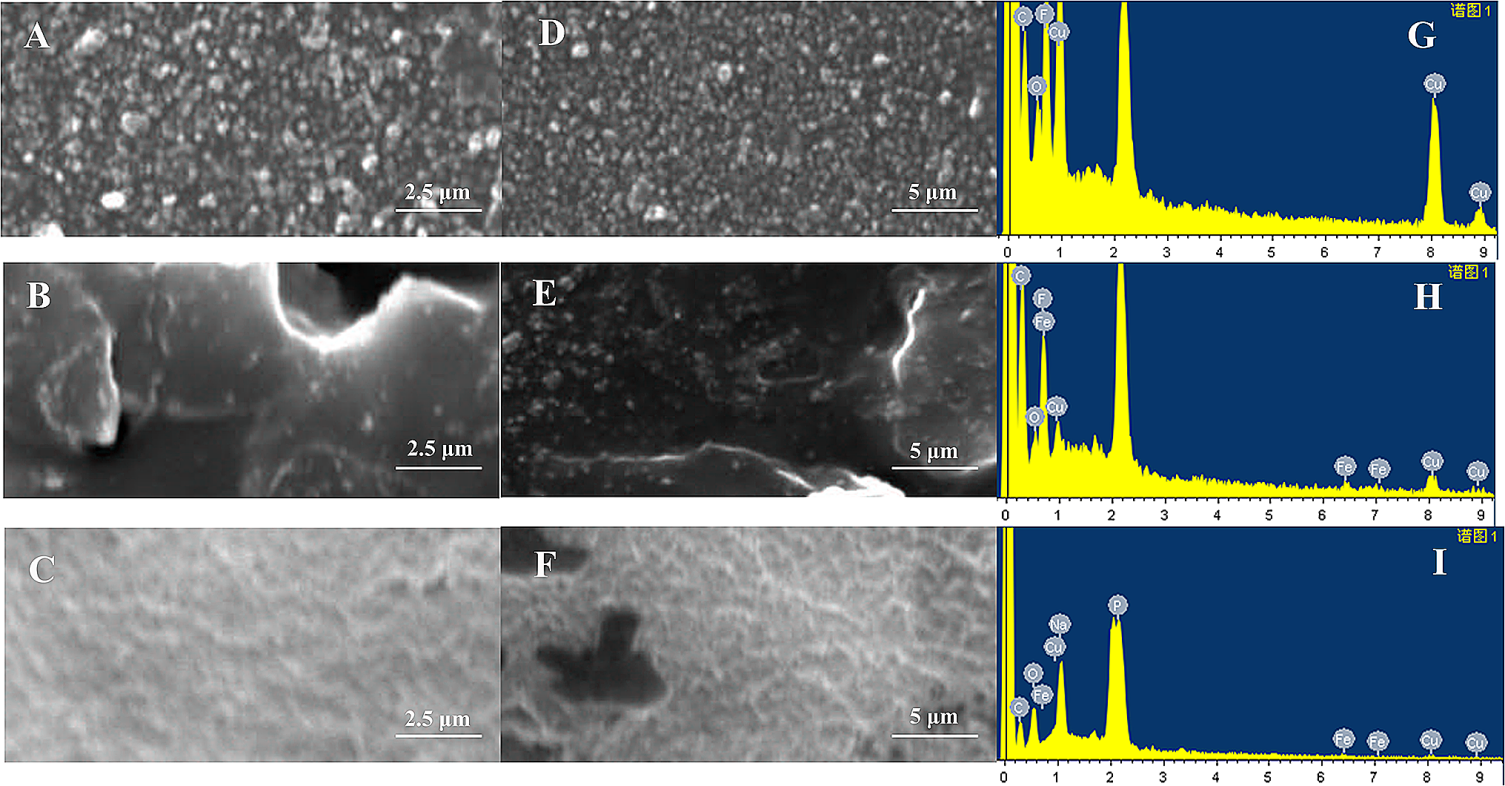
SEM characterization of different modified electrodes (**A**. **D**. Cu_2_O/GCE, **B**. **E**. Fc/Cu_2_O/GCE **C**. **F**. UOx/Fc/Cu_2_O/GCE) and energy spectrum images (**G**. Cu_2_O/GCE, **H**. Fc/Cu_2_O/GCE, **I**. UOx/Fc/Cu_2_O/GCE).

The formation and morphology of differently modified electrodes (i.e. Cu_2_O/GCE, Fc/Cu_2_O/GCE, UOx/Fc/Cu_2_O/GCE) were determined by carrying out a comparative analysis of SEM and EDS (Fig. 3). Very distinct differences in the surface structure were noted. As shown in Figs. 3A and 2D, the Cu_2_O nanoparticles was uniformly distributed on the GCE, which was favorable for the electrocatalytic reaction on the electrode surface (Fig. 3A, D). Elements such as copper, oxygen, fluorine and carbon also appeared on the energy spectrum (Fig. 3G) showed Cu_2_O was modified on GCE. When Fc was attached to the electrode, the morphology of modified electrodes gave rise to a rather rough concave and convex shape surface, and a regular porous structure was observed on the modified electrodes which indicatied Fc forms different effective electron transmission channels (Fig. 3B, E). The element iron also appeared in the corresponding energy spectrum (Fig. 3H), which illustrated Fc was modified on the Cu_2_O/GCE electrode. After the immobilization of UOx onto the rough Fc matrix surface of modified electrodes, the surface roughness decreased (Fig. 3C, F). Because conjugation of Fc and UOx can provide novel systems promoting electron transfer.^18^ In the energy spectrum sodium and phosphorus elements appeared indicating UOx in PBS buffer solution was modified on the Fc/Cu_2_O/GCE electrode (Fig. 3I). SEM and EDS results of different modified electrodes indicated that each step of electrode modification was successful.

### Electrochemical studies of prepared UOx/Fc/Cu_2_O/GCE electrode

**Figure 4:**
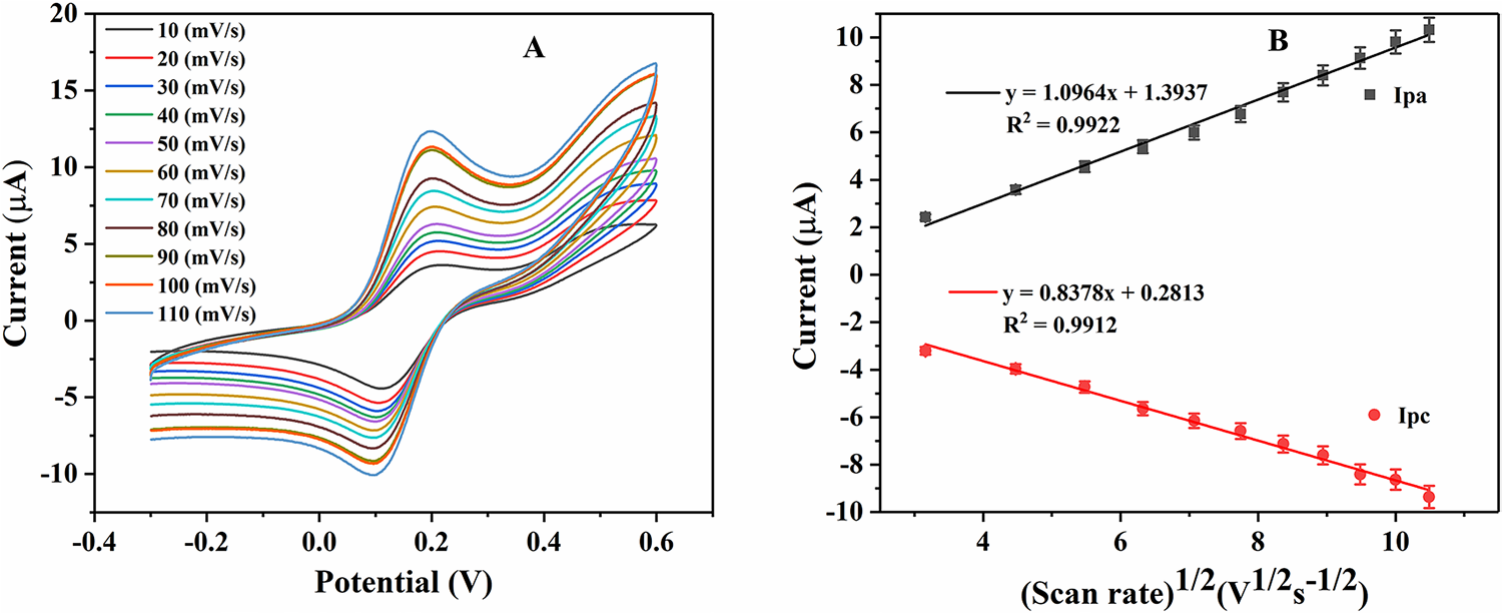
(**A**) The reaction kinetics of UOx/Fc/Cu_2_O/GCE electrode obtained by CV at scan rates from 10 to 110 mV s^−1^ in 5 mM [Fe(CN)_6_]^3-/4-^ containing 0.1 M KCl solution; (**B**) the plot of anodic/cathodic current with square root of scan rate.

The electron transfer behavior of redox couple at UOx/Fc/Cu_2_O/GCE electrodes were investigated using CV measurements in 5 mM [Fe(CN)_6_]^3-/4-^ containing 0.1 M KCl solution at different scan rates ranging from 10 to 110 mV s^−1^. The results showed (Fig. 4A) a subsequent increased in redox peak currents (Ip) (anodic (Ipa) and cathodic (Ipc) peak currents) with increasing scan rate value for UOx/Fc/Cu_2_O/GCE electrode. At 100 mV s^−1^, the peak current ratio of anodic (Ipa) and cathodic (Ipc) was about 1, which indicated that the redox quasi-reversible of the UOx/Fc/Cu_2_O/GCE electrode was good. At the same time, the increase in peak separation potential (ΔE) values due to positive shift of potential in anodic peak current (Ipa) and negative shift of potential in cathodic peak current (Ipc) with increasing scan-rate, further confirmed the quasi-reversible process taking place at the electrode surface.^31^

The redox peak currents against square root of scan rate values (ν^1/2^) were plotted in Fig. 4B. The linear fitting curve showed that the peak current had a linear relationship with the square roots of the scanning rates having regression coefficient (R^2^) of 0.9915 for Ipa and 0.9923 for Ipc respectively, which indicated the redox process to be a diffusion controlled electrochemical process.^32,33^

At the same time, the blank CV curve of UOx/Fc/Cu_2_O/GCE in 0.1 M KCl were detected. The results (Fig. S3) showed that the blank CV curve of UOx/Fc/Cu_2_O/GCE at scan rates from 10 to 100 mV s^−1^ in 0.1 M KCl has a subsequent increased in redox peak currents (Ip) (anodic (Ipa) and cathodic (Ipc) peak currents) with increasing scan rate value for UOx/Fc/Cu_2_O/GCE electrode. The linear fitting curve showed that the peak current had a linear relationship with the square roots of the scanning rates having regression coefficient (R^2^) of 0.9984 for Ipa and 0.9908 for Ipc respectively. It is speculated that ferrocene plays a certain role. But the peak currents were small compared with Fig. 4A. We can know from the results like ferrocene, [Fe(CN)_6_]^3-/4-^ had the function of increasing peak current.

**Figure 5:**
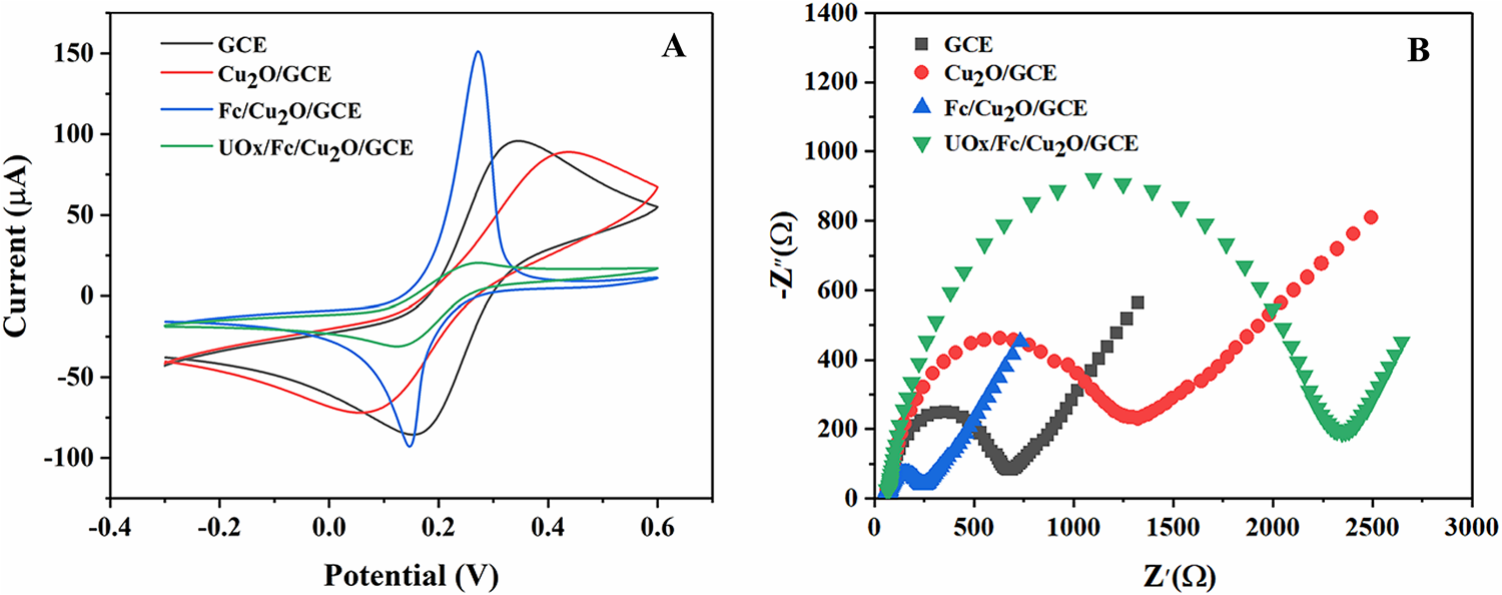
The electron-transfer capabilities of electrodes at each modification step in 5 mM [Fe(CN)_6_]^3-/4-^ containing 0.1 M KCl solution (pH = 7.0) using (**A**) CV at the scan-rate of 50 mV s^−1^ and (**B**) EIS technique respectively.

Further, CV technique was used to analysis the electrochemical behaver of different modified electrodes. Figure 5A showed a comparative analysis of CV curves obtained at (1) bare-GCE (2) Cu_2_O/GCE (3) Fc/Cu_2_O/GCE and (4) UOx/Fc/Cu_2_O/GCE at a fixed scan-rate of 50 mV/s in 5 mM [Fe(CN)_6_]^3-/4-^ containing 0.1 M KCl solution (pH = 7.0). A pair of well-defined redox peaks was observed when bare GCE was measured (curve bare-GCE), which was assigned to the reversible redox behavior of ferricyanide ion. Compared with bare GCE, the cathodic and anodic peak currents decreased slightly after the modification of Cu_2_O on the surface of GCE using Naf-adhesive (curve Cu_2_O/GCE), because the “blocking” of the electrode due to repulsion of the sulphonic acid head group of the Naf surface^21^ although Cu_2_O with excellent conductivity could accelerate electron transfer. In contrast, a pair of strong redox peaks appeared when the Fc deposited Cu_2_O/GCE electrode confirming the enhanced charge transport phenomenon on the deposited Fc matrix (curve Fc/Cu_2_O/GCE). Fc has stable chemical properties and excellent redox reversibility, which can react with the active center of biological enzymes and rapidly improve the density of response current. However, a pair of decrease redox peaks was also observed after the immobilization of UOx onto the matrix surface through covalent attachment using Glutaraldehyde: BSA cross-linker (curve UOx/Fc/Cu_2_O/GCE), which was due to the non-conducting nature of UOx and cross-linker that hinders the electron transfer process. At the same time, which indicating UOx was successfully immobilized on Fc/Cu_2_O/GCE electrode. Therefore, the fabrication of UOx/Fc/Cu_2_O/GCE electrode was completely successful.

The electron transfer properties and interfacial properties of surface modified electrode at each immobilization steps were further investigated using EIS technique. The size of the semicircular diameter of the impedance curve is proportional to the electron transfer resistance (Ret). Figure 5B showed the EIS plots of four different modified electrodes. The Ret values were found to be 2,355.14 ± 2.56, 1,286.17 ± 1.69, 676.71 ± 1.25 and 245.72 ± 1.37 Ω for UOx/Fc/Cu_2_O/GCE, Cu_2_O/GCE, bare-GCE and Fc/Cu_2_O/GCE electrodes respectively. When Cu_2_O/Naf was modified onto the bare GCE, the size of the semicircular diameter of the impedance curve enlarged, and the Ret increased (curve Cu_2_O/GCE) with respect to the bare GCE (curve GCE), because the “blocking” of the sulphonic acid head group of the Naf. Subsequently, when Fc was attached to the electrode, the semicircular diameter significantly reduced, which indicated the Ret decreased and the Fc could accelerate the electron transfer (curve Fc/Cu_2_O/GCE). Furthermore, the UOx and Glutaraldehyde: BSA modified electrode had a larger semicirclediameter than (curveUOx/Fc/Cu_2_O/GCE) which demonstrated the Ret was further increased because the biological active substances UOx and cross-linker could retard the interfacial electron transfer and decrease the accessibility of electrochemical probe. The EIS results validated the formation of different layers on the electrode surface and were consistent with those of CV investigations, thus confirmed the successful fabrication of the UOx/Fc/Cu_2_O/GCE electrode.

### Detection of UA using UOx/Fc/Cu_2_O/GCE electrode

**Figure 6:**
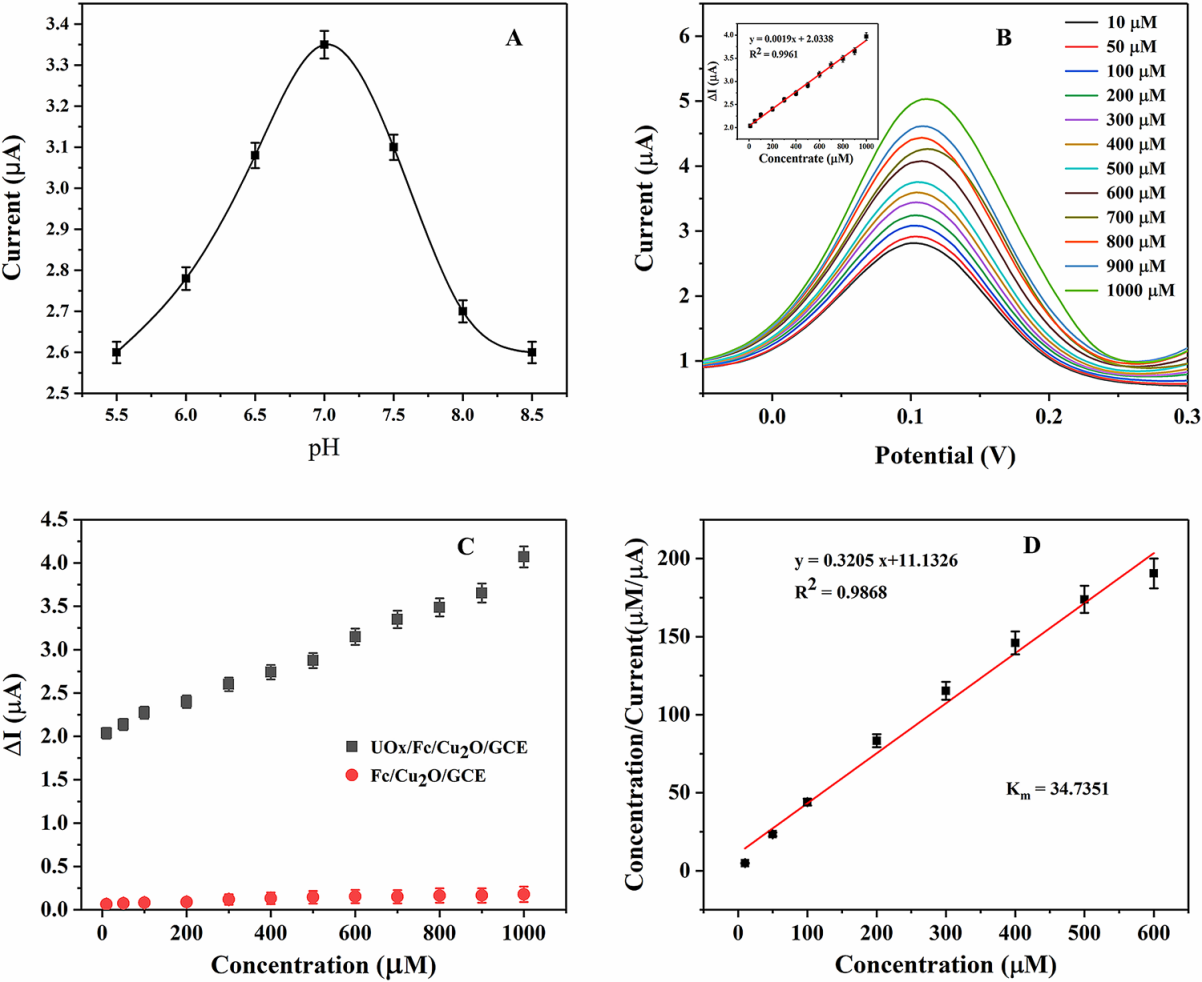
(**A**) The effect of varied pH value of the solution on the oxidation peak, (**B**) DPV response of UOx/Fc/Cu_2_O/GCE biosensor towards UA (10–1,000 μM) in 5 mM[Fe(CN)_6_]^3-/4-^ containing 0.1 M KCl solution (pH 7.0) at a scan rate of 50 mV/s. [Inset shows current versus concentration plot]. (**C**) Plot showing change in current values obtained with respect to different concentrations of UA using UOx/Fc/Cu_2_O/GCE and enzyme-free Fc/Cu_2_O/GCE electrode (i.e. control set). (**D**) Hannes plot for Michaelis–Menten constant (Km).

The pH values of electrolyte and substrate concentration have great influence on the enzyme biosensor. Therefore, we optimized these two conditions. Figure 6A showed the effect of varied pH value in a fixed concentration of UA (i.e. 200 μM). The peak current increased obviously with increasing the pH from 5.5 to 7.0, when the pH value was increased further up to 8.5 the peak current began to decrease. Therefore, pH 7.0 was selected as the best pH conditions.

**Figure 7:**
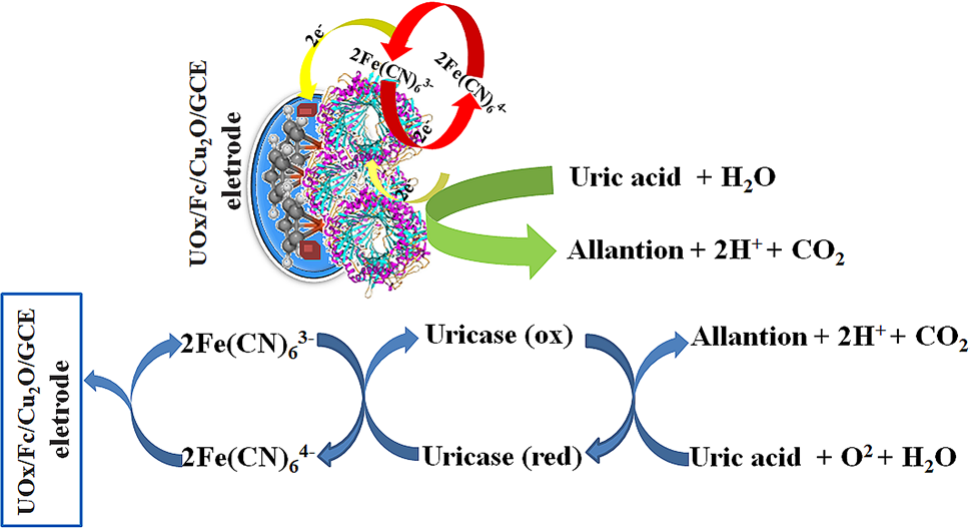
The mechanism of uric acid detection by redox medium [Fe(CN)_6_]^3-/4-^.

DPV was used to investigate the response of the modified UOx/Fc/Cu_2_O/GCE electrode towards an increasing concentration of UA (0.1–1,000 μM) in 5 mM [Fe(CN)_6_]^3-/4-^ containing 0.1 M KCl solution at a scan rate of 50 mV s^−1^, the results were shown in Fig. 6B. The biosensor indicated rapid, stable and increasing current responses over the entire concentration range. The current values increased when UA concentration was increased at the electrode. Its occurrence was due to the higher electronic availability on the electrode surface, which increases the availability of catalytic oxidation of uricase in the presence of a medium. With the presence of a medium [Fe(CN)_6_]^3-/4-^, the current value of catalytic oxidation of uric acid increases with the increase of the concentration of uric acid on the electrode. Electrons were transferred from the uricase to the electrode surface by redox media, resulting in direct transfer of electrons between them. The mechanism of uric acid detection by redox medium [Fe(CN)_6_]^3-/4-^ was illustrated in Fig. 7. Uricase is continuously catalytic oxidized to allantoinn, and it was reduced which causes a decrease in uricase concentration. Ferricyanide ions existed in electrochemical cells, which extracted electrons from the redox center of reductase molecules to regenerate the enzyme into an activation form. Ferricyanide ions in this process were reduced to ferricyanide ions. Hence, the ions concentration of ferricyanide near the electrode surface was increased, which showed that the concentration of oxidized UA was in direct proportion to the increase of concentration of ferricyanide ion. The increased ferrocyanide ions were oxidized under the applied potential, and electrons were released on the electrode surface, resulting in the increase of peak current.

The peak current of the UOx/Fc/Cu_2_O/GCE electrode was linear to UA concentration within the range of 0.1–1,000 μM. The linear regression equation was y = a + bx, where y was the peak current (Ip, μA), and x was the UA concentration (μM) with an R squared correlation of 0.9900 (inset image of Fig. 6B). According to the calibration curve of current and concentration, the sensitivity of biosensor was 1.900 μA mM^−1^ cm^−2^.

Use the formula 3 SD/sensitivity to calculate the detection limit of UA^1^, where SD was the standard deviation obtained from the current measured in blank (without UA), which was 0.0377, So the detection limit was 0.0596 μM. The significant sensitivity and detection limit of the prepared biosensors were much lower than the physiological range, which may be due to the expected synergistic effect of Fc electron mediator and Cu_2_O NPs decorated UOx that enables convenient signal conduction and effective electrocatalytic activity.

The kinetics of the immobilized UOx was calculated using the apparent Michaelis–Menten constant (Km) using Hanes Plot.^1^ At the same time, the electrode (Fc/Cu_2_O/GCE) without enzyme modification was used to detect different concentrations of UA as a negative control to verify the important role of UOx in the determination of UA (Fig. 6C). The results showed that there was only a small amount of current and the peak current values of different concentrations of substrate showed irregular changes compared with those of enzyme modified electrode (UOx/Fc/Cu_2_O/GCE) detection. Therefore, it can be intuitively speculated that UA can be detected by UOx-modified electrode, which can lead to a large number of regular changes in the current, and then it can be detected sensitively. Hanes plot was constructed between [UA conc.]/current and [UA conc.] as shown in Fig. 6D. The straight line regression equation was y = 0.3205x + 11.1326 with regression coefficient of 0.9868. A very low magnitude Km values were evaluated as 34.7351 μM which indicated the chemical attraction of the enzyme towards the uric acid was much higher (the lower the Km value, the higher the substrate affinity).

### Reproducibility, stability and specificity studies

The stability of the enzyme based biosensor was examined by repeating measurements of 200 μM UA. The biosensor showed almost identical response up to 35 successive measurements with relative standard deviation (RSD) of 3.2% indicating its good reusability. The reproducibility of the biosensor was investigated from the response to 200 μM UA at three electrodes fabricated by uniform procedure. The biosensor showed the high reproducibility with a RSD of 2.8%.

**Figure 8:**
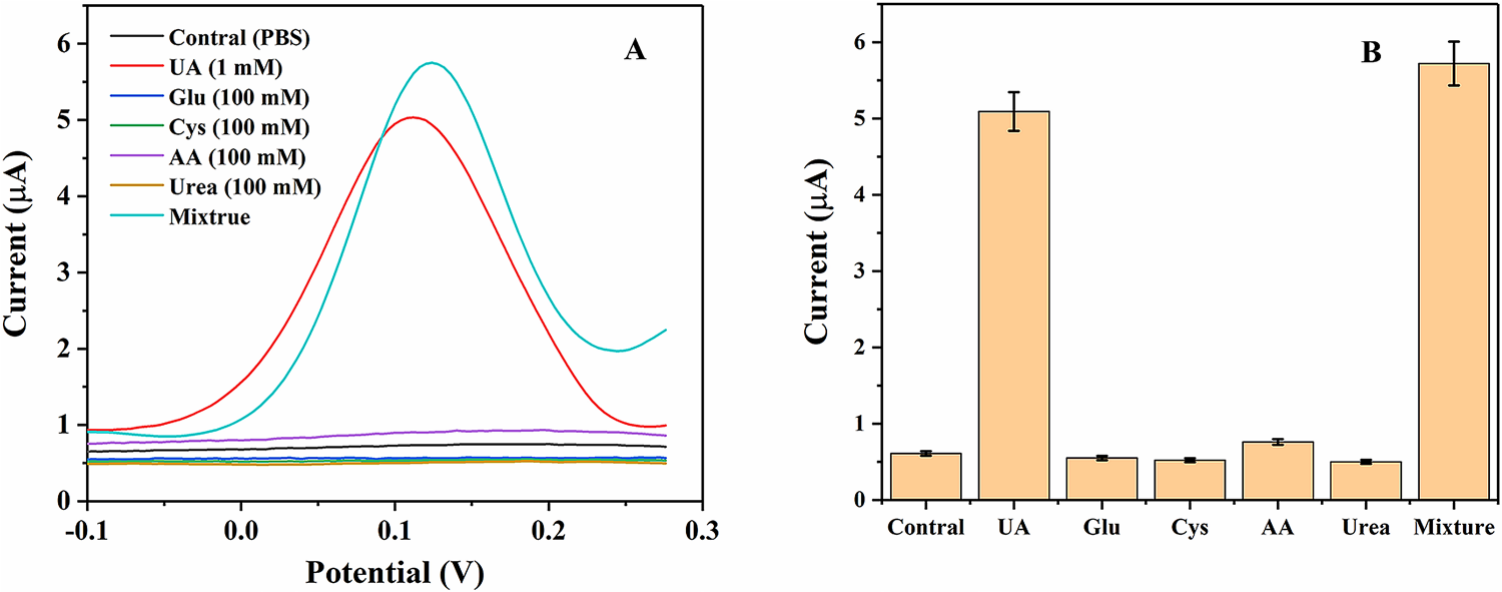
Selectivity test for fabricated biosensor with different interfering analytes (**A**) DPV (each 100 mM and UA 1 mM) in 5 mM [Fe(CN)_6_]^3-/4-^ containing 0.1 M KCl solution (pH 7.0).

To investigate the specificity of the biosensor towards UA, the effect of some commonly found interferents in real samples such as glucose(Glu), cystein (Cys), ascorbic acid (AA) and urea was determined by the fabricated biosensor in 5 mM [Fe(CN)_6_]^3-/4-^ containing 0.1 M KCl solution (pH 7.0), the selectivity of the biosensor was measured in the presence of 100 mM of each glucose (Glu), cystein (Cys), ascorbic acid (AA) and urea respectively. 1 mM of UA and a mixture of all the interferents with UA (1 mM) was also analysed (Fig. 8). The signal of other interfering compounds was approximately same as that of blank solution and the signal corresponding to UA and the mixture was the same (Fig. 8A), which showed no interaction of these biomolecules. The control bar here, refers to the signal corresponding to the 5 mM [Fe(CN)_6_]^3-/4-^ containing 0.1 M KCl solution (pH 7.0) (blank; without any interferents) (Fig. 8B). The results showed the fabricated biosensor had strong anti-interference ability.

### Analysis of real samples

Three normal human urine samples were detected with the UOx/Fc/Cu_2_O/GCE biosensor to verify its application for real samples. Standard addition method was used to detect the UA content in the urine samples. The urine samples containing a series of known concentrations (100, 200, 300, 400 and 500 μM) of UA were measured and calculated according to the response current with the standard calibration curve. The % recovery value for each spiked concentration was calculated as per the previous literatures.^33,34^ The % recovery values for the spiked samples were 93.36–102.06% suggesting the reliability of the existing biosensor and its efficiency towards UA analysis in clinical detection (Table 1). In Table 1, Cs was concentration of analyte spiked into the sample. Co was the concentration of analyte in unspiked sample. Ci was the total concentration of analyte in the spiked samples estimated from the calibration plot. Recovery = (Ci − Co) * 100/Cs.

**Table 1:** Determination of UA concentrations in spiked urine samples.

The content of UA	Spiked UA concentration Cs (μM)	Recovered UA concentration Ci–Co (μM)	Recovery (%) (n = 6)	Relative standard deviation (RSD) % (n = 6)
289.06	100	93.36	93.36	1.02
	200	202.04	101.02	0.98
	300	288.79	96.26	0.99
	400	408.24	102.06	1.15
	500	489.99	98.00	1.21

### Comparison of biosensor performance

Table 2 showed the performance of the fabricated sensor in comparison to earlier UA biosensors reported in literatures. From Table 2, we can know the detection range be wider than the other sensors and the sensitivity was higher. The detection limit was much lower compared with that of other biosensors. Most important of all, our system had the lower Km value and hence the sensor’s affinity towards its substrate UA was the higher.

**Table 2:** Comparison of biosensor performance.

Matrix	Detection range	Sensitivity	LOD	Km	References
Uricase/NiO/Pt/Ti/glass	0.05–1 mM	1,278.48 µM mM^−1^	0.11 mM	0.17 mM	^1^
Uricase/AuNPs/MWCNTs/Au	0.01–0.8 mM	0.44 mA mM^−1^	0.01 mM	0.5 mM	^35^
Uricase/CuO/Pt/glass	0.05–1 mM	2.7 mA mM^−1^	0.14 mM	0.12 mM	^36^
Nafion/Uricase/ZnO/Au	0.1–0.59 mM	89.74 μA mM^−1^ cm^−2^	25.6 μM	0.99 mM	^37^
Uricase/Prussian blue/SPE	0.03–0.3 mM	–	0.01 mM	–	^38^
Nafion/Uricase/Ferrocene/GCE	0.5–600 μM	1.78 μA μM^−1^	230 nM	14.07 µM	^21^
L-Met-FcAld/Au/Cu	0–2.38 mM	72.8 μA mM^−1^	2.4 μM	–	^39^
Uricase/Au-rGO/ITO	50–800 μM	86.62 ± 0.19 μA mM^−1^	7.32 ± 0.21 μM	51.75 µM	^34^
UOx/EDC:NHS/CZTS/ITO	0–700 μM	1.838 µA µM^−1^ cm^−2^	0.066 µM	13 µM	^2^
UOx/Fc/Cu_2_O/GCE	0.01–1 mM	1.900 μA mM^−1^ cm^−2^	0.0596 μM	34.7351 µM	This work

## Conclusions

The Cu_2_O NPs and stable entrapment of chemical activator (Fc) in the protein matrix for enhancing biocatalytic activity of an enzyme was used to modify GCE and the amplified biocatalytic activity of UOx showed a significant electrocatalytic activity toward the detection of UA. The results revealed that the prepared biosensor could successfully detect UA at wide concentration range of 0.1–1,000 μM with fairly low detection limit of 0.0596 μM, and a lower Km value of 34.7351 μM. At the same time, it exhibited good stability and specificity towards UA in presence of non-target analytes which confirms it high potential for efficient detection of UA that can be extended for use in clinical settings.

## Supplementary information

Supplementary information

## References

[1] Arora K, Tomar M, Gupta V (2011). Highly sensitive and selective uric acid biosensor based on RF sputtered NiO thin film. Biosens. Bioelectron..

[2] Jain S, Verma S, Singh SP, Sharma SN (2019). An electrochemical biosensor based on novel butylamine capped CZTS nanoparticles immobilized by uricase for uric acid detection. Biosens. Bioelectron..

[3] Nery, E. W. Analysis of Glucose, Cholesterol and uric acid. In *Analysis of samples of clinical and alimentary interest with paper-based devices.* Springer theses (recognizing outstanding Ph.D. research) (Springer, Cham, 2016).

[4] Rock KL, Kataoka H, Lai JJ (2013). Uric acid as a danger signal in gout and its comorbidities. Nat. Rev. Rheumatol..

[5] Nyhan WL (1997). The recognition of Lesch–Nyhan syndrome as an inborn error of purine metabolism. J. Inherit. Metab. Dis..

[6] Swinson D, Snaith J, Buckberryc J, Brickley M (2010). High performance liquid chromatography (HPLC) in the investigation of gout in palaeopathology. Int. J. Osteoarchaeol..

[7] Rocha DL, Rocha FRP (2010). A flow-based procedure with solenoid micro-pumps for the spectrophotometric determination of uric acid in urine. Microchem. J..

[8] Jin D (2016). Quantitative determination of uric acid using CdTe nanoparticles as fluorescence probes. Biosens. Bioelectron..

[9] Zhang Y (2016). Separation and characterization of bufadienolides in toad skin using two-dimensional normal-phase liquid chromatography × reversed-phase liquid chromatography coupled with mass spectrometry. J. Chromatogr. B.

[10] Dai H, Nan W, Wang D, Zhang X, Ma H, Meng L (2016). Voltammetric uric acid sensor based on a glassy carbon electrode modified with a nanocomposite consisting of polytetraphenylporphyrin, polypyrrole, and graphene oxide. Microchim. Acta..

[11] Hou C, Liu H, Zhang D, Zhang M (2016). Synthesis of ZnO nanorods-Au nanoparticles hybrids via in-situ plasma sputtering-assisted method for simultaneous electrochemical sensing of ascorbic acid and uric acid. J. Alloys Compd..

[12] Zhao J, Mu F, Qin L, Jia X, Yang C (2015). Synthesis and characterization of MgO/ZnO composite nanosheets for biosensor. Mater. Chem. Phys..

[13] Gaia R (2016). Enzyme biosensors for biomedical applications: strategies for safeguarding analytical performances in biological fluids. Sensors.

[14] Vijayakumar AR, Csoregi E, Heller A, Gorton L (1996). Alcohol biosensors based on coupled oxidase-peroxidase systems. Anal. Chim. Acta..

[15] Dong S, Wang B, Liu B (1992). Amperometric glucose sensor with ferrocene as an electron transfer mediator. Biosens. Bioelectron..

[16] Erden PE, Kılıç E (2013). A review of enzymatic uric acid biosensors based on a mperometric detection. Talanta.

[17] Hu Y, Du W, Chen C (2014). Fabrication of flower-shaped Pt-Au-graphene nanostructure and its application in electrochemical detection of glucose. Chin. J. Anal. Chem..

[18] Sneed BT (2014). Shaped Pd-Ni-Pt Core-sandwich-shell nanoparticles: influence of Ni sandwich layers on catalytic electrooxidations. ACS Nano.

[19] Wang K, Dong X, Zhao C, Qian X, Xu Y (2015). Facile synthesis of Cu_2_O/CuO/RGO nanocomposite and its superior cyclability in supercapacitor. Electrochim. Acta..

[20] Khan R (2014). Glucose-assisted synthesis of Cu_2_O shuriken-like nanostructures and their application as nonenzymatic glucose biosensors. Sens. Actuat. B Chem..

[21] Ghosh T, Sarkar P, Turner APF (2015). A novel third generation uric acid biosensor using uricase electro-activated with ferrocene on a Nafion coated glassy carbon electrode. Bioelectrochemistry.

[22] Herrick RS (1996). Ordered conformations in bis(amino acid) derivatives of 1,1′-ferrocenedicarboxylic acid. Tetrahedron Lett..

[23] Zhao M, Zhao J, Qin L, Jia H, Liu S (2019). Synthesis of Ta/Ni microcavity array film for highly sensitive uric acid detection. J. Electroanal. Chem..

[24] Chen M, Wei X, Qian H, Diao G (2011). Fabrication of GNPs/CDSH-Fc/nafion modified electrode for the detection of dopamine in the presence of ascorbic acid. Mater. Sci. Eng. C Mater..

[25] Ye SR, Rathmell AR, Wilson AR, Wiley BJ (2014). Copper nanowires: the role of cuprous oxide seeds in the one-pot and seeded syntheses of copper nanowires. Small.

[26] Surikanti GR, Bajaj P, Sunkara MV (2019). g-C_3_N_4_-mediated synthesis of Cu_2_O To obtain porous composites with improved visible light photocatalytic degradation of organic dyes. ACS Omega.

[27] Li FX (2020). Promoting the spatial charge separation by building porous ZrO_2_@TiO_2_ heterostructure toward photocatalytic hydrogen evolution. J. Colloid Interface Sci..

[28] Fu Y (2020). In-situ chemical vapor deposition to fabricate cuprous oxide/copper sulfide core–shell flowers with boosted and stable wide-spectral region photocatalytic performance. J. Colloid Interf. Sci..

[29] Wang JC (2015). Enhanced photoreduction CO_2_ activity over direct Z-scheme alpha-Fe_2_O_3_/Cu_2_O heterostructures under visible light irradiation. ACS Appl. Mater. Interface.

[30] Zhang J, Yu JG, Zhang YM, Li Q, Gong JR (2011). Visible light photocatalytic H_2_-production activity of CuS/ZnS porous nanosheets based on photoinduced interfacial charge transfer. Nano. Lett..

[31] Allen JB, Larry RF (2001). Electrochemical Methods Fundamentals and Applications.

[32] Jindal K, Tomar M, Gupta V (2013). Nitrogen-doped zinc oxide thin films biosensor for determination of uric acid. Analyst.

[33] Verma S (2017). Anti-IL8/AuNPs-rGO/ITO as an immunosensing platform for noninvasive electrochemical detection of oral cancer. ACS Appl. Mater. Interfaces.

[34] Verma S, Choudhary J, Singh KP, Chandra P, Singh SP (2019). Uricase grafted nanoconducting matrix based electrochemical biosensor for ultrafast uric acid detection in human serum samples. Int. J. Biol. Macromol..

[35] Chauhan N, Pundir CS (2011). An amperometric uric acid biosensor based on multiwalled carbon nanotube-gold nanoparticle composite. Anal. Biochem..

[36] Jindal K, Tomar M, Gupta V (2012). CuO thin film based uric acid biosensor with enhanced response characteristics. Biosens. Bioelectron..

[37] Zhao Y, Yan X, Kang Z, Lin P, Fang X (2013). Highly sensitive uric acid biosensor based on individual zinc oxide micro/nanowires. Microchim. Acta..

[38] Piermarini S, Migliorelli D, Volpe G, Massoud R, Palleschi G (2013). Uricase biosensor based on a screen-printed electrode modified with Prussian blue for detection of uric acid in human blood serum. Sens. Actuat. B Chem..

[39] Cheng C, Kao C (2016). An electrochemical biosensor with uricase immobilized on functionalized gold coated copper wire electrode for urinary uric acid assay. Electroanal..

