# Supplementary material for: A highly sensitive uric acid electrochemical biosensor based on a nano-cube cuprous oxide/ferrocene/uricase modified glassy carbon electrode
Source: Sci Rep. 2020 Jun 30;10:10607. doi: 10.1038/s41598-020-67394-8 (PMC7327035; doi:10.1038/s41598-020-67394-8)
Supplement: Supplementary file 1 — Supplementary information [file 41598_2020_67394_MOESM1_ESM.pdf]

A Highly Sensitive Uric Acid Electrochemical Biosensor Based on a Nano-cube Cuprous Oxide/Ferrocene/Uricase Modified Glassy Carbon Electrode

Qinghua Yan[1] ·Na Zhi[1] ·Li Yang[2*] ·Guangri Xu[2] ·Qigao Feng[2] ·Qiqing Zhang[1, 3] ·Shujuan Sun[4]

[1] *The Key Laboratory of Biomedical Material, School of Life Science and Technology, Xinxiang Medical University, Xinxiang, 453003, China*

[2] *School of Chemistry and Chemical Engineering, Henan Institute of Science and Technology, Xinxiang 453003, China*

[3] *Institute of Biomedical Engineering, Chinese Academy of Medical Sciences & Peking Union Medical College, Tianjin, 300192, China*

[4] *The Hospital of Eighty-third Group Army，Xinxiang，453000，China*

* Li Yang. Email: xkbhome@163.com Tel: 86-373-3831887 Fax: 86-373-3831887

Qinghua Yan e-mail: yqh3499@163.com

Na Zhi e-mail: 2281652902@qq.com

Guangri Xu e-mail: guangrix@163.com

Qigao Feng e-mail: qigaof@163.com

Qiqing Zhang e-mail: zhangqiq@126.com

Shujuan Sun e-mail: 252776013@qq.com

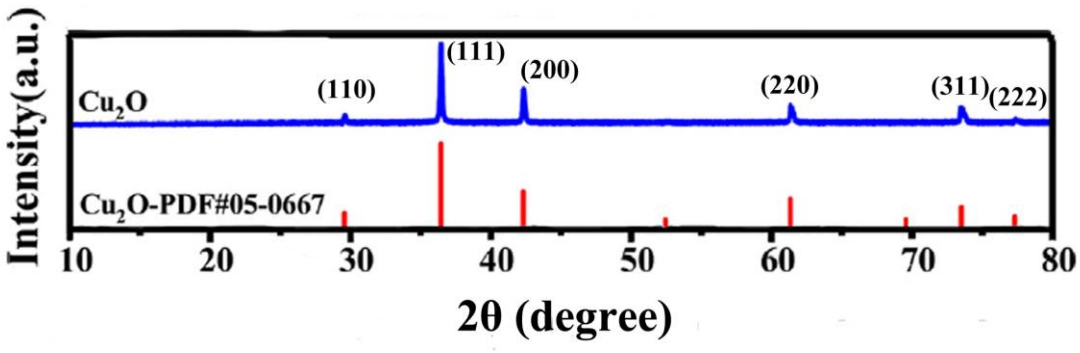

Fig. S1 Powder x-ray diffraction patterns of Cu_2O NPs was compared with the standard pattern of cuprous oxide cubic crystal system (PDF#05-0667)

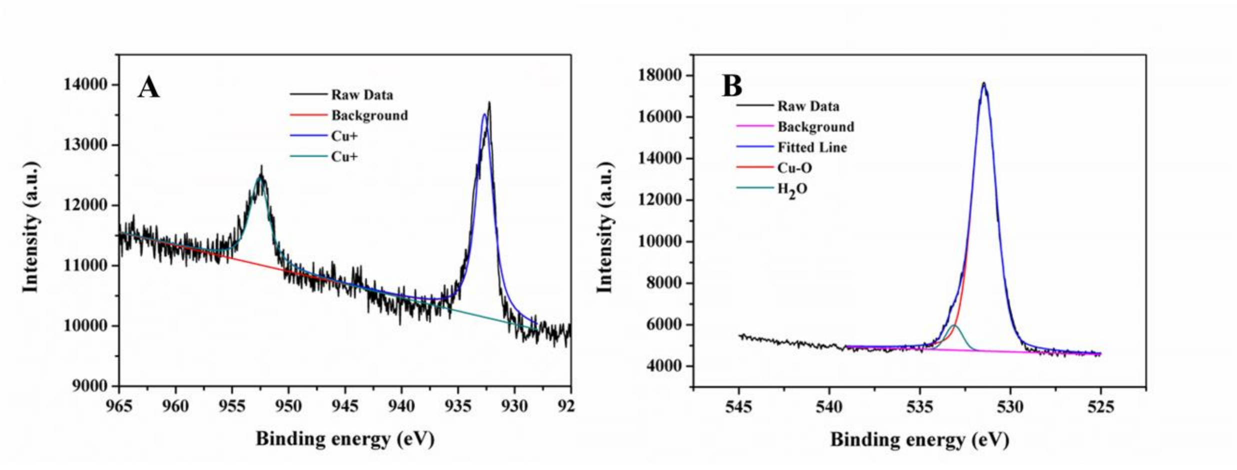

Fig. S2 XPS spectrum of Cu_2O NPs.

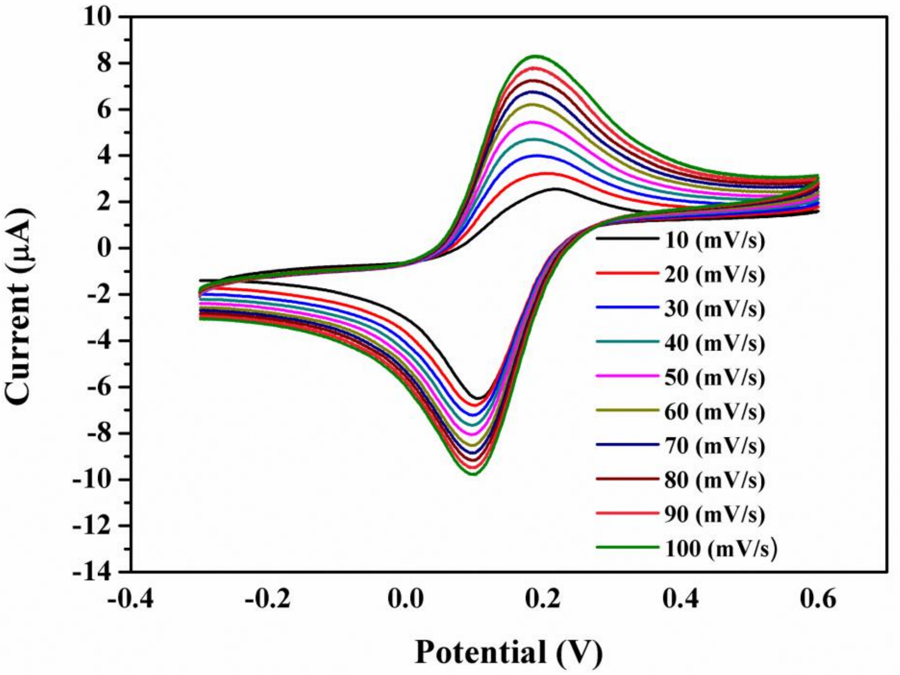

Fig. S3 The reaction kinetics of UOx/Fc/Cu_2O/GCE electrode obtained by CV at scan rates from 10 to 110 mV s^{-1}in 0.1 M KCl solution
